# Burden and predisposing factors of physical inactivity among adults in Africa: Systematic review and Meta-analysis

**DOI:** 10.1371/journal.pone.0348786

**Published:** 2026-05-11

**Authors:** Aychew Kassa Belete, Bantie Getnet Yirsaw, Birhan Ambachew Taye

**Affiliations:** 1 Faculty of Natural and Computational Science, Department of Sport Science‌‌, Woldia University, Woldia, Ethiopia; 2 Faculty of Natural and Computational Science, Department of Statistics, Woldia University‌‌, Woldia, Ethiopia; 3 Department of Epidemiology and Biostatistics, Institute of Public Health, College of Medicine and Health Sciences, University of Gondar, Gondar, Ethiopia; Ministry of Health, General Health Directorate of Raparin and University of Raparin, IRAQ

## Abstract

**Introduction:**

Physical inactivity is a primary driver of global mortality, data on its specific impact in Africa remains fragmented. We conducted this study to provide the first comprehensive pooled prevalence and identify predisposing factors contributing to physical inactivity among African adults.

**Method:**

We searched PubMed, Scopus, Embase, and Google Scholar for studies published between March 1, 2010, and March 31, 2025. A quality assessment of the studies was performed through the Newcastle-Ottawa Scale (NOS). The random-effect (DerSimonian) model was used to calculate the aggregated rates of physical inactivity among adults and their predisposing factors. Heterogeneity was assessed using the I^2^ statistic, along with subgroup and sensitivity analyses. Publication bias was evaluated using Egger’s test, and all analyses were performed in STATA, version 17.

**Results:**

This systematic review and meta-analysis included 34 studies with a total of 41,521 participants. The pooled prevalence of physical inactivity was 45% (95% CI: 35%–55%), and there was considerable heterogeneity (I^2^ = 98.4%). The highest prevalence of 57% (95% CI: 0.33, 0.81) I^2^ = 97.7 seen in 2013 and the lowest prevalence 28% (95% CI: 0.03, 0.53) I^2^ = 94.3%) observed in 2011. This study identified that, being female (OR = 1.87; 95% CI: 1.55, 2.25), Adults aged ≥ **60**, (OR=2.18; 95% CI:1.98,4.77), being obese (OR=3.52; 95% CI: 2.26,5.49), urban dweller (OR=1.91, 95% CI:1.24,2.95), being overweight (OR=2.03; 95% CI: 1.61,2.68), depression symptom (OR= 1.34;95% CI: 1.07,1.68) and more drinking of alcohol (OR=1.73; 95% CI: 1.18,2.64), were significant predisposing factors associated with prevalence of physical inactivity.

**Conclusion:**

A high level of physical inactivity was observed amongst the African adults. Our findings indicate that female gender, older age, being overweight and obese, high levels of alcohol consumption and depression are significant predictors of physical inactivity in the African population. Promoting awareness about the positive impacts of regular exercise on health among women and an elderly age group could effectively lower the chances of hypokinetic diseases.

## Introduction

Physical inactivity, defined as engaging in less than 600 Metabolic Equivalent of Task (MET) minutes per week of activity, is a major health risk factor [1]. Currently, setting is considered as the new smoking, meaning prolonged sitting and sedentary behavior have serious health consequences like smoking [2]. Despite the WHO’s advice on physical activity for adults 18 years and older, the majority of the world’s population is sedentary [3]. It is the fourth leading cause of death globally, responsible for an estimated 3.2 million deaths annually, of which 2.6 million are in developing countries (WHO, 2009, 2010) [4]. Also, physical inactivity has been approximated to cost the global healthcare system $538 billion each year [5]

Physical activity refers to any movement which requires energy expenditure and is caused by skeletal muscle [6]. It covers both intended physical activity and physical activity which forms part of a person’s everyday activities such as driving, work, and maintenance. it is a form of physical activity, such as cycling, dancing, swimming, walking, jogging, gardening, and sports. Aside from boosting energy expenditure, the activities have been proven to enhance overall health [7]. In this regard, adults 18–64 years should take at least 150–300 minutes of moderate-intensity aerobic physical activity each week, 75–150 minutes of vigorous-intensity aerobic physical activity each week, or an equivalent level of both. The adults must do at least two days of strength exercise activity each week involving their major muscle groups [8].

In 2020, the World Health Organization initiated the Global Action Plan on Physical Activity (GAPPA) 2018–2030 with the aim to reduce global physical inactivity by 15% by the year 2030 [9]. Recent data from the WHO, however, show slow progress towards the same. Currently, nearly a third of adults worldwide are not physically active enough [10]. The global age-standardized rate of physical inactivity has risen from 26.4% in 2010 to 31.3% in 2022 [11]. The highest was seen in the high-income Asia Pacific (48.1%) [12], followed by South Asia (45.4%) [13]. The lowest was reported in Oceania (13.6%) [14], followed by sub-Saharan Africa (SSA) at 16.8% [15].

In African region, physical inactivity is typically high and linked with urbanization, transportation shifts, and the uptake of less active lifestyles [16]. Inactivity levels are extremely variable across countries, ranging from 8% (Kenya) to 70% (Egypt) not meeting the WHO’s minimum physical activity levels.

Furthermore, results from several studies indicate that socio-demographic factors like gender [17,18–22], age [17,19,20,23], marital status [19,20,23,24], place of residence [20,22,25], education level [26,24], body mass index [19,21,27,28] and behavioral factors such as, depression levels [28–30], history of alcohol use [31,27,28,30], and work status have been reported to influence physical activity levels significantly [32–34]. Understanding these socio-demographic and behavioral factors of physical inactivity can support planning for targeted prevention and intervention initiatives and identifying more effective health promotion strategies.

Physical inactivity survey largely bases its results on self-observations and the use of standardized questionnaires such as global physical activity questionnaire (GPAQ) and international physical activity questionnaire (IPAQ). Although objective measures are used through such devices as actigraphy in some studies, most other studies use self-reports, which are cheaper. The three sampling strategies that are supposed to be used to sample participants normally involve random sampling to ensure bias is eliminated, stratified sampling to get a representation of the subgroups, and convenience sampling which is more prone to bias but it is the simplest to undertake. All these methodologies have the advantage of providing a comprehensive data on physical inactivity in various populations. This study will contribute largely to the achievement of Sustainable Development Goal 3 with target 3.4 specifically whose objective is to reduce premature deaths due to non-communicable diseases by one-third through prevention and treatment, with mental health and wellbeing improvement [35]. However, so far, no comprehensive study has been made using systematic review and meta-analysis to determine prevalence of physical inactivity and its determinants among adults in Africa. Therefore, the objective of this study was to establish the pooled prevalence and the factors that are contributing to physical inactivity in this region.

## Methods

### Study protocol and registration‌‌

This study was conducted on the prevalence of physical inactivity and predisposing factors among adults in Africa, and conducted following guidelines of the Preferred Reporting Items for Systematic Reviews and Meta-Analysis (PRISMA). The study has been registered under the registration number CRD420251023946 in the International Prospective Register of Systematic Reviews (PROSPERO). Ethical approval and participant consent were not applicable for this study, as it utilized secondary data from published literature and involved no human or animal interventions.

### Searching strategies and sources of information

We conducted a search for studies published between March 1, 2010, and March 31, 2025 using electronic databases including Scopus, PubMed, Embase‌‌, and google scholar. Keywords like Prevalence, Magnitude, Physical inactivity, insufficient physical activity, determinants, and Africa with their corresponding Medical Subject Headings (MeSH) terms were used to search by combining using Boolean operators (AND, OR, NOT). For instance, the advanced PubMed search strategy was: (“physical activity” OR “physical inactivity” OR “insufficient physical activity” OR “sedentary behavior” [MESH Terms] OR “exercise” [MESH Terms]) AND (Lesotho OR Swaziland OR Botswana OR Namibia OR South Africa OR Angola OR Cameroon Equatorial Guinea OR Gabon OR Congo OR Chad OR Central African Republic OR Congo the Democratic Republic Sao Tome and Principe OR Burundi OR Eritrea OR Madagascar OR Reunion OR Somalia OR Comoros OR Ethiopia OR Rwanda OR Djibouti OR Kenya OR Mayotte OR Seychelles OR Uganda OR Mozambique OR Zambia OR Malawi OR Tanzania, Zimbabwe OR Benin OR Liberia OR Saint Helena OR Burkina Faso OR Gambia OR Mali OR Ghana OR Mauritania OR Senegal OR Cape Verde OR Cote D’ivoire OR Guinea OR Niger OR Sierra Leone OR Guinea-Bissau OR Nigeria OR Togo OR Algeria OR Egypt OR Libyan Arab Jamahiriya OR Morocco OR Tunisia OR Western Sahara OR Sudan) Filters applied: from 01/03/2010–31/03/2025(S1 Table).

### Article selection and eligibility criteria

For this systematic review and meta-analysis, we included the following types of papers: full text articles, abstracts, and thesis or dissertations that were written only in English, all observational studies that report the prevalence of physical inactivity, preprint and peer-reviewed articles the publication year between March 1, 2010 to March 31, 2025 were included.

Duplicate studies, research done in languages other than English, review articles, and studies conducted before March 1, 2010 and after March 31, 2025 were not included in this analysis.

### Study selection and quality assessment

Two reviewers (BAT and BGY) independently assessed study quality using the Newcastle-Ottawa Scale (NOS), with a third reviewer (AKB) resolving any discrepancies. Studies were evaluated based on sample representativeness, size, non-response rate, outcome ascertainment, and comparability. Quality was categorized as excellent (8–9), very good (6–7), good (4–5), or poor (< 4). To ensure high standards, only studies scoring at least 8 out of 9 points were included in the final analysis, while those scoring below 4 were excluded (S2 Table).

### The outcome of the study

The key goal of the study was to determine the overall prevalence of physical inactivity among the adults in Africa, and the secondary goal was to find out what factors lead to physical inactivity in this population.

### Data extraction process

The data were independently extracted by three authors (AKB, BGY, and BAT) using a Microsoft Excel spreadsheet. The data extracted included: The author’s name, publication year, study setting or country, study period, study design, sample size, prevalence of physical inactivity, data collection period, and adjusted odds ratio (AOR) with a 95% confidence interval for predisposing factors of prevalence of physical inactivity among adults in Africa were extracted.

### Handling missing data, author contact protocol, and inter rater reliability measures

Managing missing data is very important in meta-analysis, as researchers look at its effects using multiple imputation and sensitivity analyses. When a protocol is used to connect with the authors, we can record the information and actions taken as well as the schedule for contacting them. Also, to ensure all writers interpret data consistently, Cohen’s kappa or interclass correlation coefficients are used to measure the reliability.

### Statistical analysis

The random-effect (DerSimonian) model was applied to calculate how frequent physical inactivity is among adults in Africa and what might influence it. Research heterogeneity was assessed with the I^2^ statistic and to find out where it might be coming from, subgroup analysis was used. To determine the robustness of the overall estimates sensitivity analyses were applied. This included the process of verifying how the presence or lack of certain set of studies, especially those that revealed a great discovery, or that which could be considered an outlier, had an effect on the findings. The degree of diversity from study to study was measured by I^2^ and markers of low, medium and high heterogeneity were defined as 25%, 50% and 75%, respectively [36]. To spot publication bias, the Egger test was applied and p-values lower than 0.05 showed that it was significant [37]. Asymmetry was found in the funnel plot and with Egger’s test which led to the trim-and-fill method being used to re-calculate the pooled effect size by removing extreme effect sizes. This extra prevalence was then included again in the funnel plot to estimate the most accurate pooled prevalence. The analysis was carried out with STATA version 17 statistical software.

## Results

### Search outcomes and characteristics of included studies

**Fig 1:**
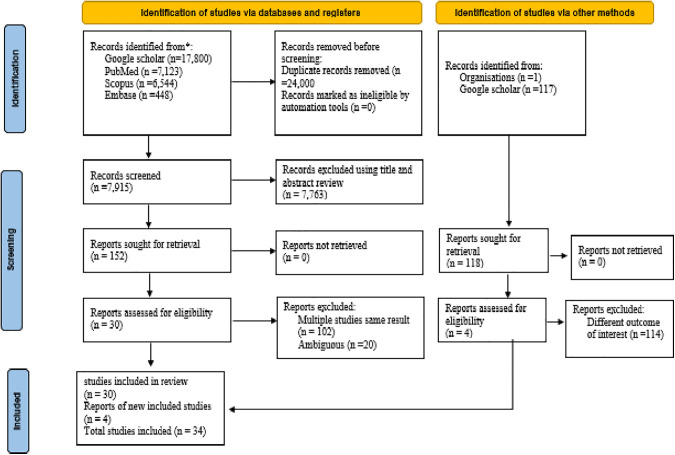
PRISMA flow diagram for new systematic reviews which included searches of databases, registers and other sources for physical inactivity among adults in Africa‌‌.

From the total 32,033 recorded studies. We detected 31,915 from databases and 118 from other sources. Out of the 31,915 studies that were found through database searches, 24,000 papers were eliminated prior to screening for duplication, 7,882 from titles and abstract reviews, 20 from ambiguity, and 102 from numerous reports of the same outcome. Out of the 118 studies that were found using alternative approaches, 114 of them were deemed irrelevant. Finally, 34 studies were included for systematic and meta-analysis (Fig 1).

### Characteristics of included studies

A total of 34 studies were included to estimate the pooled prevalence of physical inactivity. The studies were conducted in whole African countries between 2010 and 2025. The minimum number of adults who participated in the single study was 205 and the maximum number was 9801 (Table 1).

**Table 1:** Summary statistics for the number of adults in the study the prevalence of physical inactivity among adults in Africa.

Author	Year	Country	Design	Sample Size	Prevalence
Usman et al.	2015	Nigeria	cross-sectional	1048	31.40
Abdul et al.	2018	Tanzania	cross-sectional	2311	65
Kirunda	2017	Uganda	cross-sectional	1210	37.60
Ononamadu et al.	2016	Nigeria	case-control study	912	69.41
Ukegbu et al.	2022	Nigeria	case-control study	868	50
Roba et al.	2029	Ethiopia	case-control study	872	45.10
Solomon et al.	2022	Ethiopia	cross-sectional	641	29.48
Gelibo et al.	2017	Ethiopia	cross-sectional	9801	5.80
Owoaje et al.	2013	Nigeria	cross-sectional	525	27.40
Ogbonna et al.	2013	Nigeria	cross-sectional	205	90.70
Ayodele et al.	2010	Nigeria	cross-sectional	1000	38
Osho et al.	2013	Nigeria	cross-sectional	305	43.30
Ajayi et al.	2022	Nigeria	cross-sectional	440	48.40
Adegoke and Oyeyemi 2011	2011	Nigeria	cross-sectional	1006	41
Nigussie et al.	2023	Ethiopia	cross-sectional	308	30.50
Madukwe et al.	2013	Nigeria	cross-sectional	2983	64.20
Asiki et al.	2018	Kenya	cross-sectional	4500	7.7
Salako et al.	2010	Nigeria	cross-sectional	2000	3.29
Onajole et al	2012	Nigeria	cross-sectional	400	92
Bısırıyu et al.	2017	Nigeria	cross-sectional	1560	32.5
Teklemariam et al.	2018	Ethiopia	cross-sectional	601	45.50
Yalew et al.	2024	Ethiopia	cross-sectional	838	65.60
Chiroma et al.	2015	Nigeria	cross-sectional	498	19.2
Oyeyemi and Adeyemi 2013	2013	Nigeria	cross-sectional	292	58.56
Akanbi et al.	2017	Nigeria	cross-sectional	883	77.80
Abd El et al	2019	Egypt	cross-sectional	850	14
Farrag et al.	2011	Egypt	cross-sectional	319	15.4
El-Gilany etal.	2019	Egypt	cross-sectional	671	71
Nketiah et al	2023	Ghana	cross-sectional	219	81.7
Andersen et al.	2021	Algeria	cross-sectional	355	43
Ali	2022	Somalia	cross-sectional	217	43.3
Oyeyemi et al.	2015	Nigeria	cross-sectional	934	69
Yousif et al.	2019	Sudan	cross-sectional	216	45
Malambo et al.	2016	south Africa	cross-sectional	1733	34

Regarding the study design, thirty-one studies were cross sectional and three studies were case-control studies. Concerning publication year, from a total of 34 studies, 2 [38,39] were in 2010, 2 [21,40] were in 2011, 1 [41] in 2012, 5 [42,30,43–45]in 2013, 3 [46–48]in 2015, 2 [49,50]in 2016, 4 [20,23,29,51] in 2017,3 [26,24,52] in 2018, 4 [53,54,18,25] in 2019, 1 [55] in 2021,4 [56,19,27,57] in 2022, 2 [58,22] in 2023, and 1 [28] in 2024, In the case of Counties 17(50%) of studies were conducted in Nigeria, 6 (17.6%) in Ethiopia, 3 (8.8%) in Egypt, and 1 (2.9%) in Algeria, Ghana, Kenya, Somalia, South Africa, Sudan, Tanzania, and Uganda each country

### Pooled prevalence of physical inactivity among adults in Africa

**Fig 2:**
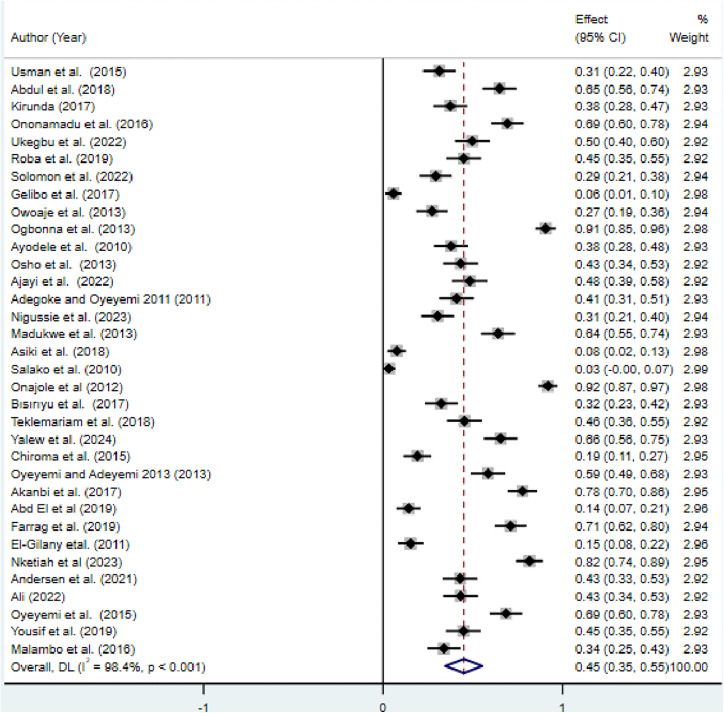
Forest plot for pooled prevalence of physical inactivity among adults in Africa.

As Fig 2 showed from a total of 34 studies, a total of 41521 adults were included, and the pooled magnitude of physical inactivity among adults was found to be 45% (95% CI: 0.35–0.55), with observed heterogeneity (I^2^ = 98.4%; p-value < 0.001) (Fig 2).

### Source of heterogeneity and handling

#### Sub-group analysis.

Subgroup analysis was performed using the year of publication, country, and study design. (Table 2) two studies were published in each year 2010,2011, 2016 and 2023 separately, five study in 2013, four in 2017, and 2022 separately, and three in 2015, and 2018, single studies in 2012,2021, and 2024 with the highest prevalence of 57% (95% CI: 0.33,0.81) I^2^ = 97.7 seen in 2013 and the lowest prevalence 28% (95% CI: 0.03, 0.53) I^2^ = 94.3%) observed in 2011. Here, the highest heterogeneity was observed in studies conducted in 2010 (I^2^ = 97.8%). (S1 Fig)

**Table 2:** Subgroup analysis for the year of publication, country, and study design prevalence of physical inactivity among adults in Africa region.

Subgroup analysis		Number of studies	Pooled prevalence of Physical inactivity	I^2^	p-value
Publication year	2010	2	20(0.14,0.54)	97.8%	0.000
	2011	2	28(0.03,0.53)	94.3	0.000
	2012	1	92(0.87,0.97)	0.0%	
	2013	5	57(0.33,0.81)	97.7%	0.000
	2015	3	40(0.11,0.69)	97.1%	0.000
	2016	2	52(0.17,0.86)	96.5%	0.000
	2017	4	36(0.05,0.72)	98.7%	0.000
	2018	3	39(0.02,0.76)	96.5%	0.000
	2019	4	44(0.18,0.69)	97.1%	0.000
	2021	1	43(0.33,0.53)	0.0%	
	2022	4	43(0.33,0.52)	74.3%	0.009
	2023	2	56(0.06,1.06)	98.6%	0.000
	2024	1	66(0.56,0.75)	0.0%	
	**Total**	**34**	**45(0.35,0.55)**	**98.4%**	**0.000**
Country	Algeria	1	43(0.33,0.53)	0.0%	
	Egypt	3	34(0.0,0.67)	98.3%	0.000
	Ethiopia	6	37(0.17,0.56)	97.2%	0.000
	Ghana	1	82(0.74,0.89)	0.0%	
	Kenya	1	8(0.02,0.13)	0.0%	
	Nigeria	17	50(0.34,0.67)	98.8%	0.000
	Somalia	1	43(0.34, 0.53)	0.0%	
	south Africa	1	34(0.25,0.45)	0.0%	
	Sudan	1	45(0.35,0.55)	0.0%	
	Tanzania	1	65(0.56,0.74)	0.0%	
	Uganda	1	38(0.28,0.47)	0.0%	
	**overall**	**34**	**45(0.35,0.55)**	**98.4%**	**0.000**
Study design	Case-control	3	44(0.33,0.55)	98.5%	0.000
	Cross-sectional	31	55(0.40,0.70)	86.5%	0.00
	**Total**	**34**	**45(0.35,0.55)**	**98.4**	**0.000**

**Note:** (*I*^2^ = 0.0%: implies there was a single study in that specific category so that *I*^2^ was not calculated),

Regarding study design, the highest prevalence 55% (95% CI: 0.40, 0.70) I^2^ = 86.5%) was observed under cross-sectional study, and 44% (95% CI: 0.33, 0.55) I^2^ = 98.5%) were under case- control studies. Based on study design highest heterogeneity (I^2^ = 98.5%) was observed studies conducted using case-control study designs. (S2 Fig)

In the case of Counties, 17 studies were conducted in Nigeria 50% ((95% CI: 0.34,0.67) I2 = 98.8%), 6 studies in Ethiopia 37% (95% CI: 0.17,0.56) I^2^ = 97.2%), 3 studies in Egypt 34% (95% CI: −0.0,0.67) I^2^ = 98.3%), and single studies in Algeria 43% (95%CI: 0.33,0.53) I^2^ = 0.0%), Ghana 82% (95% CI: 0.74,0.89) I^2^ = 0.0%), Kenya 8% (95% CI: 0.02,0.13) I^2^ = 0.0%), Somalia 43% (95% CI: 0.34, 0.53) I^2^ = 0.0%), South Africa 34% (95% CI: 0.25,0.45) I^2^ = 0.0%), Sudan45% (95% CI: 0.35,0.55) I^2^ = 0.0%), Tanzania 65% (95%CI: 0.56,0.74) I^2^ = 0.0%), and Uganda 38% (95% CI: 0.28,0.47) I^2^ = 0.0%) each country. Here the highest prevalence 50%(95% CI: 0.34,0.67) I^2^ = 98.8% was observed studies that were conducted in Nigeria and 34% (95% CI: (−0.0,0.67) I^2^ = 98.3% of prevalence was for studies conducted in Egypt. As results showed the highest heterogeneity was observed for studies that were conducted in Nigeria. (S3 Fig)

### Sensitivity analysis

**Fig 3:**
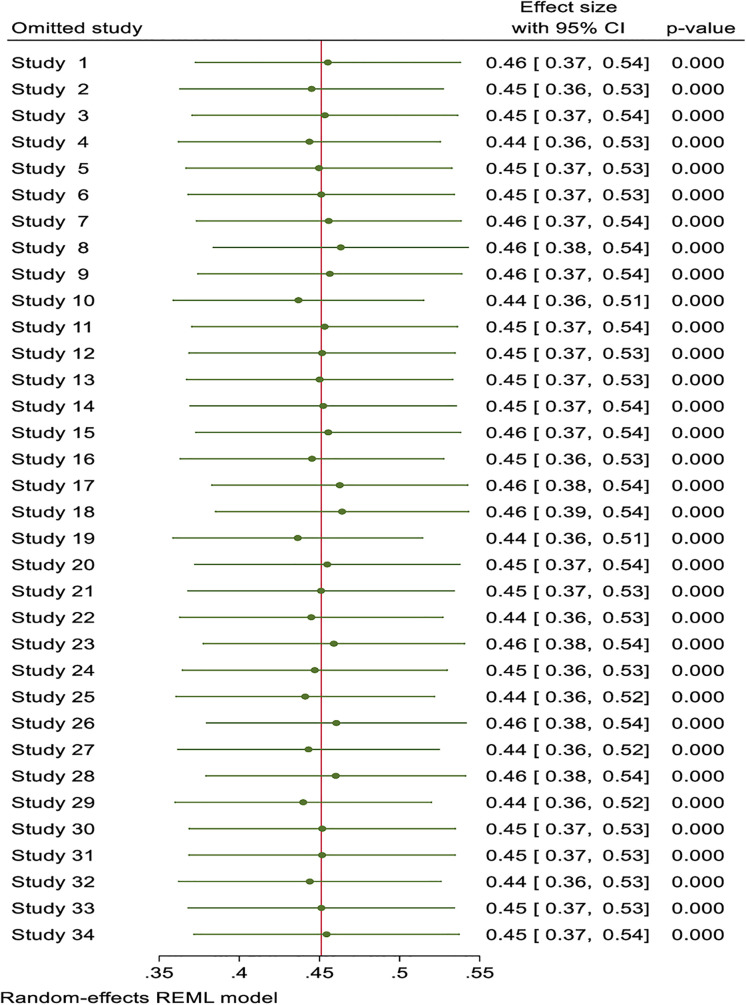
Sensitivity analysis for prevalence of physical inactivity among adults in Africa.

Sensitivity analysis was conducted, and the result showed that there is no single study whose value lies outside the 95% CI of the overall estimate or pooled prevalence of physical inactivity among adults (Fig 3)

### Publication bias

**Fig 4:**
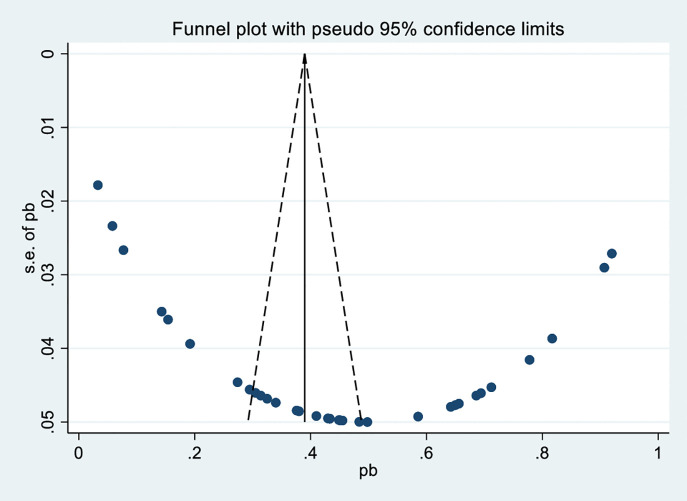
Funnel plot of prevalence with standard error.

To evaluate whether there are small study effects, or publication bias when it comes to the studies, the Egger test was applied, and the funnel plot was obtained. The funnel plot (Fig 4) indicates the asymmetry of the distribution of the data points with the higher number on the right-hand side, and the pseudo 95% confidence limits are not symmetric, indicating the possible publication bias in favor of large and positive effect sizes. But to prove that this bias is here and the level of its influence an egger test is required

Egger’s regression analysis indicates a significant positive slope and negative bias, implying smaller studies report lower effect sizes. The low p-value provides strong evidence against the null hypothesis of no small-study effects, suggesting publication bias may be present in this meta-analysis (Table 3).

**Table 3:** Eager test.

Std_Eff	Coefficient	Std. err.	T	P > t	[95%conf. interval]	
slope	.0000934	.158209	0.00	1.000	−.3221677	.3223545
bias	10.60925	4.103474	2.59	0.014	2.250746	18.96775

**Fig 5:**
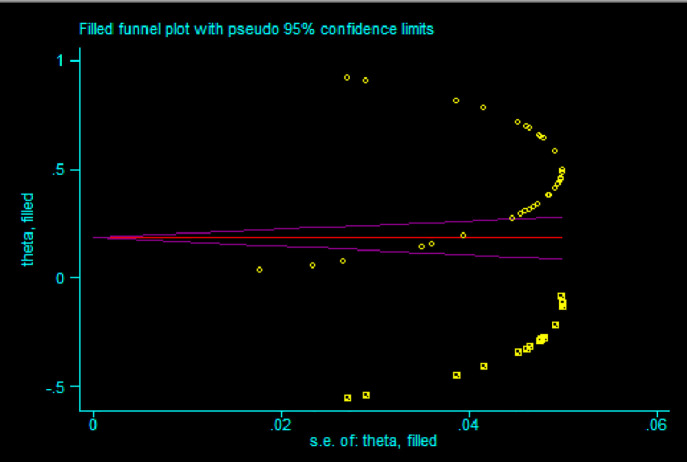
Trim and fill plot.

Among the factors affecting publication bias are journals choosing to promote findings with big results, potential prejudices among editors and experts and researchers changing data to achieve noticeable results. By using the funnel plot and Egger’s test, asymmetry was discovered, so the trim-and-fill method (Fig 5) was applied to exclude outliers and re-calculate the correct pooled effect size.

### Pooled predisposing factors of physical inactivity among adults in Africa

This table (Table 4) reports the pooled odds ratio for studies that investigated two or more factors connected to adults’ low levels of physical activity. Therefore, adult’s prevalence of physical inactivity is significantly associated with Gender, Age, Marital status, residence, Categories of BMI, depression and alcohol consumption

**Table 4:** Summary of pooled odds ratio for factors associated with Physical inactivity among adults.

Predispozig factor	Catagorie	No of studies	Pooled odds ratio	P-value	I^2^
Sex	Female	12	1.87(1.55,2.25)	<0.0001	68.7%
	Male (reference)				
Age	40-49	6	1.61(0.74,3.50)	0.56	93.9%
	50-59	6	1.21(0.68,2.15)	0.91	91.0%
	≥ 60	6	2.18(1.98,4.75)	<0.0001	90.7%
	18–39(reference)				
Marital status	Married	5	1.24(0.87,1.77)	0.85	76.4%
	Divorced	5	1.26(0.95,1.67)	0.57	58.9%
	Widowed	5	1.56(1.21,2.01)	0.002	79.0
	Single (reference)				
Educational status	Primary	8	1.12(0.41,3.06)	0.73	98.0%
	Secondary	8	0.66 (0.05, 7.73)	0.701	97.1%
	Collage and above	8	1.34(0.67,2.67)	0.08	92.5%
	No formal edu (refe)				
Occupational status	Employed	4	1.12(0.70, 1.79)	0.54	84.2%
	Unemployed (refe)				
Residence	Urban	4	1.91(1.24,2.95)	0.003	78.0%
	Rular (refe)				
Body compostion	Underweight	4	0.99(0.58,1.69)	0.89	52.3%
	Overweight	4	2.03 (1.61, 2.68)	<0.0001	67.6%
	Obese	4	3.52 (2.26, 5.49)	<0.0001	60.0%
	Normal(reference)				
Depression	Yes	3	1.34 (1.07, 1.68)	0.007	87.2%
	No (refe)				
Alchol consumption	No	4	1.73 (1.18, 2.64)	<0.001	64.5%
	Yes(refe)				

Adults being female were 1.87 times more likely to be physically inactive than adults who being male (OR = 1.87 (95% CI: 1.55, 2.25), I^2^ = 68.7%). Adults who are aged more than 60 were 2.18 times more likely to physically inactive than adults age between 18–39 (OR=2.18(95% CI:1.98,4.75, I^2^ = 90.7). Adults who are being widowed had 1.56 times higher odds, than those of single adults. Regarding the place of residence, adults who were from urban settings were 1.91 times more likely to physically inactive than rural residents (OR=1.91(95% CI:1.24,2.95), I^2^ = 78.0%). Interms of body composition, adults who were being overweight had 2.03 times higher odds than adults who are being normal body mass index (OR=2.03(95% CI: 1.61, 2.68), I^2^ = 67.6%). In addition, adults who are being obese had 3.52 times higher odds than normal adults. As for depression, adults who are have depression had 1.34 times more likely to physically inactive than adults who are free from depression (OR=1.34(95% CI: 1.07, 1.68), I^2^ = 87.2%). Regarding to alchole consumption, adults who are drinking alchole had 1.73 times higher odds of physical inactivity compared to adults who are not drink alchole (OR=1.73(95% CI: 1.18, 2.64), I^2^ = 64.5%).

## Discussion

This study was intended to determine the pooled prevalence of physical inactivity and its associated factors among adults in Africa. Our systematic and meta-analysis used a total of thirty-four eligible studies with 41,521 adults and found that pooled prevalence of physical inactivity was 45% (95% CI: 0.35–0.55). The pooled prevalence rate was higher than studies conducted in India 36.7% [59], and Brazil 41.1% [60].This finding is close to the study conducted in Malaysia 43.7% [59] and Ethiopia 45.5% [24], On the other hand, the pooled prevalence was lower than a study conducted in China, 56.2% [61] Ethiopia 65.6% [28], This difference may be due to differences in participant characteristics, attention given by the government about physical activity, study design, cultural and behavioral practices, geographical area, difference in publication year, and difference in applying sampling techniques.

Regarding to gender of the adults, women were more likely to be physically inactive than adults who are being male (AOR = 1.87; 95% CI: 1.55, 2.25). This is supported by the study done in US [62], and Switzerland [63]. The possible explanation might be due to understanding the societal, psychological, and environmental factors that contribute to these differences. Factors such as social norms and expectations, which often dictate gender roles, may limit women’s opportunities for physical activity, while disparities in access to recreational facilities and safe exercise environments further exacerbate this issue. Additionally, psychological factors like body image concerns, motivation, and self-efficacy may differ between genders, influencing their engagement in physical activity [64]. These results are consistent with previous research suggesting that women often face unique barriers to maintaining an active lifestyle [65].

Older adults were consistently associated with a higher odds of physical inactivity compared to younger adults. Adults who are aged more than 60 years were more likely to physically inactive than adults age between 18–39 years (AOR = 2.18; 95% CI: 1.98, 4.75). This trend can be attributed to several factors, including physical limitations, chronic health conditions, and a decrease in overall mobility that often accompany aging. Additionally, older adults may experience barriers such as lack of access to safe exercise environments, limited social support, and reduced motivation to engage in physical activity.

Adults who are being widowed had 1.56 times higher odds, than those of single adults (AOR = 1.56; 95% CI: 1.21, 2.01). Adults from urban settings are more likely to be physically inactive than their rural counterparts due to a combination of environmental, social, and psychological factors (AOR = 1.91; 95% CI: 1.24, 2.95). This finding is supported by a study [66,67]. Urban areas often have higher population densities, leading to increased reliance on cars and fewer safe spaces for outdoor activities. The fast-paced urban lifestyle can create time constraints and stress, reducing motivation for exercise. While urban environments may provide more recreational facilities, barriers such as cost and overcrowding can hinder access. In contrast, rural communities often promote physical activity through stronger social ties and communal events. Addressing these disparities requires targeted interventions that consider the unique challenges faced by urban populations.

Interms of body composition, adults who were being 0verweight had 2.03 times higher odds than adults who are being normal body mass index residents (AOR = 2.03; 95% CI: 1.61, 2.68). Inaddition, adults who are being obese had 3.52 times higher odds and those of being normal body mass index (AOR = 3.52; 95% CI: 2.26, 5.49). Obese adults tend to have higher odds of physical inactivity compared to those with a normal weight due to several interrelated factors.This result supported by a study conducted in USA [68,69]. Excess weight can lead to physical limitations such as joint pain, fatigue, and decreased mobility, making physical activity more challenging and less enjoyable. Additionally, psychological factors play a significant role; obesity is often associated with low self-esteem, anxiety, and depression, which can diminish motivation to engage in physical activity. Social stigma and discrimination may further discourage participation in exercise, as obese individuals may feel embarrassed or isolated in active settings.

As for depression, adults who have depression had 1.34 times more likely to physically inactive than adults who are free from depression (AOR = 1.34; 95% CI: 1.07, 1.68). This heightened likelihood of inactivity can be attributed to several interconnected factors. Depression often leads to fatigue, low motivation, and feelings of worthlessness, which can diminish the desire to engage in physical activities.This result is supported by a study [70].

Regarding to alchole consumption, adults who are drinking alchole had 1.73 times higher odds of physical inactivity compared to adults who are not drink alchole (AOR = 1.73; 95% CI: 1.18, 2.64). This association may stem from several factors, including the sedative effects of alcohol, which can lead to fatigue and reduced motivation for physical activity. This result is in line with studies conducted by [71,72]

This study has its own strengths and weaknesses. This study has several methodological strengths that enhance its reliability. We employed a comprehensive literature search to minimize selection bias and established rigorous inclusion criteria to focus on high-quality studies. Additionally, we utilized robust statistical methods for data analysis, ensuring accurate synthesis of findings. These approaches contribute to the credibility of our pooled prevalence estimates of physical inactivity among adults. Among the limitations of this study; firstly, studies written other than in English were ignored, so studies conducted in other languages were missed. Secondly, high heterogeneity was observed. In addition, we cannot ensure that, for instance, the 17 studies conducted in Nigeria included 100% unique participants. This was an indication that the prevalence was different across studies due to study design, geographical region, population, and study year. A random effect model (DerSimonian) and subgroup analysis were conducted to overcome this problem. Thirdly, publication bias was observed due to the researchers of individual studies manipulating data in order to determine significant findings. We used the trim and fill plot procedure to identify the best estimate of the unbiased pooled effect size.

## Conclusion and recommendation

Physical inactivity is notably widespread among older adults in Africa. Factors such as being female, advancing age, lower educational attainment, unemployment, symptoms of depression, and excessive alcohol consumption were linked to higher rates of inactivity. We recommend promoting accessible, community-based physical activity programs tailored for older adults, particularly women and the unemployed. Public health efforts should also integrate mental health support to address the link between depression and physical inactivity.

## Supporting information

S1 Table Searching Strings of different data base. (DOCX)

S2 Table Quality assessment for individual studies using Newcastle Ottawa quality assessment scale. (DOCX)

S3 Table PRISMA 2020 main checklist. (DOCX)

S1 Fig Subgroup analysis by year of publication. (TIF)

S2 Fig Subgroup analysis by study design. (TIF)

S3 Fig Subgroup analysis by country. (TIF)

## Abbreviations

GAPPA: Global Action Plan on Physical Activity
CI: Confidence interval
SSA: Sub-Saharan African
NCDs: Non-Communicable Diseases
OR: Odds Ratio
WHO: World Health Organization
MET: Metabolic Equivalent of Task
