# Correction: Burden and predisposing factors of physical inactivity among adults in Africa: Systematic review and Meta-analysis

**DOI:** 10.1371/journal.pone.0352595

**Published:** 2026-06-24

**Authors:** Aychew Kassa Belete, Bantie Getnet Yirsaw, Birhan Ambachew Taye

There are errors in the author affiliations. The correct affiliations are as follows:

Aychew Kassa Belete^1^, Bantie Getnet Yirsaw^2^, Birhan Ambachew Taye^3^

**1** Faculty of Natural and Computational Science, Department of Sport Science, Woldia University, Woldia, Ethiopia, **2** Department of Epidemiology and Biostatistics, Institute of Public Health, College of Medicine and Health Sciences, University of Gondar, Gondar, Ethiopia, **3** Faculty of Natural and Computational Science, Department of Statistics, Woldia University, Woldia, Ethiopia.
