# Supplementary material for: Burden and predisposing factors of physical inactivity among adults in Africa: Systematic review and Meta-analysis
Source: PLoS One. 2026 May 11;21(5):e0348786. doi: 10.1371/journal.pone.0348786 (PMC13160333; doi:10.1371/journal.pone.0348786)
Supplement: S1 Table — (DOCX) [file pone.0348786.s001.docx]

| Data base | searching strategies |
| --- | --- |
| Pub mead | ("physical activity" OR "physical inactivity" OR "insufficient physical activity" OR "sedentary behavior" [MESH Terms] OR "exercise" [MESH Terms]) AND (Lesotho OR Swaziland OR Botswana OR Namibia OR South Africa OR Angola OR Cameroon Equatorial Guinea OR Gabon OR Congo OR Chad OR Central African Republic OR Congo the Democratic Republic Sao Tome and Principe OR Burundi OR Eritrea OR Madagascar OR Reunion OR Somalia OR Comoros OR Ethiopia OR Rwanda OR Djibouti OR Kenya OR Mayotte OR Seychelles OR Uganda OR Mozambique OR Zambia OR Malawi OR Tanzania, Zimbabwe OR Benin OR Liberia OR Saint Helena OR Burkina Faso OR Gambia OR Mali OR Ghana OR Mauritania OR Senegal OR Cape Verde OR Cote D'ivoire OR Guinea OR Niger OR Sierra Leone OR Guinea-Bissau OR Nigeria OR Togo OR Algeria OR Egypt OR Libyan Arab Jamahiriya OR Morocco OR Tunisia OR Western Sahara OR Sudan) Filters applied: from 01/03/2010–31/03/2025. |
| Embase | ("physical activity" OR "physical inactivity" OR "insufficient physical activity" OR "sedentary behavior" [MESH Terms] OR "exercise" [MESH Terms]) AND (Lesotho OR Swaziland OR Botswana OR Namibia OR South Africa OR Angola OR Cameroon Equatorial Guinea OR Gabon OR Congo OR Chad OR Central African Republic OR Congo the Democratic Republic Sao Tome and Principe OR Burundi OR Eritrea OR Madagascar OR Reunion OR Somalia OR Comoros OR Ethiopia OR Rwanda OR Djibouti OR Kenya OR Mayotte OR Seychelles OR Uganda OR Mozambique OR Zambia OR Malawi OR Tanzania, Zimbabwe OR Benin OR Liberia OR Saint Helena OR Burkina Faso OR Gambia OR Mali OR Ghana OR Mauritania OR Senegal OR Cape Verde OR Cote D'ivoire OR Guinea OR Niger OR Sierra Leone OR Guinea-Bissau OR Nigeria OR Togo OR Algeria OR Egypt OR Libyan Arab Jamahiriya OR Morocco OR Tunisia OR Western Sahara OR Sudan) Filters applied: from 01/03/2010–31/03/2025. |
| Scopus | ("physical activity" OR "physical inactivity" OR "insufficient physical activity" OR "sedentary behavior" [MESH Terms] OR "exercise" [MESH Terms]) AND (Lesotho OR Swaziland OR Botswana OR Namibia OR South Africa OR Angola OR Cameroon Equatorial Guinea OR Gabon OR Congo OR Chad OR Central African Republic OR Congo the Democratic Republic Sao Tome and Principe OR Burundi OR Eritrea OR Madagascar OR Reunion OR Somalia OR Comoros OR Ethiopia OR Rwanda OR Djibouti OR Kenya OR Mayotte OR Seychelles OR Uganda OR Mozambique OR Zambia OR Malawi OR Tanzania, Zimbabwe OR Benin OR Liberia OR Saint Helena OR Burkina Faso OR Gambia OR Mali OR Ghana OR Mauritania OR Senegal OR Cape Verde OR Cote D'ivoire OR Guinea OR Niger OR Sierra Leone OR Guinea-Bissau OR Nigeria OR Togo OR Algeria OR Egypt OR Libyan Arab Jamahiriya OR Morocco OR Tunisia OR Western Sahara OR Sudan) Filters applied: from 01/03/2010–31/03/2025. |
| Web of science | ("physical activity" OR "physical inactivity" OR "insufficient physical activity" OR "sedentary behavior" [MESH Terms] OR "exercise" [MESH Terms]) AND (Lesotho OR Swaziland OR Botswana OR Namibia OR South Africa OR Angola OR Cameroon Equatorial Guinea OR Gabon OR Congo OR Chad OR Central African Republic OR Congo the Democratic Republic Sao Tome and Principe OR Burundi OR Eritrea OR Madagascar OR Reunion OR Somalia OR Comoros OR Ethiopia OR Rwanda OR Djibouti OR Kenya OR Mayotte OR Seychelles OR Uganda OR Mozambique OR Zambia OR Malawi OR Tanzania, Zimbabwe OR Benin OR Liberia OR Saint Helena OR Burkina Faso OR Gambia OR Mali OR Ghana OR Mauritania OR Senegal OR Cape Verde OR Cote D'ivoire OR Guinea OR Niger OR Sierra Leone OR Guinea-Bissau OR Nigeria OR Togo OR Algeria OR Egypt OR Libyan Arab Jamahiriya OR Morocco OR Tunisia OR Western Sahara OR Sudan) Filters applied: from 01/03/2010–31/03/2025. |
| Google scholar | ("physical activity" OR "physical inactivity" OR "insufficient physical activity" OR "sedentary behavior" [MESH Terms] OR "exercise" [MESH Terms]) AND (Lesotho OR Swaziland OR Botswana OR Namibia OR South Africa OR Angola OR Cameroon Equatorial Guinea OR Gabon OR Congo OR Chad OR Central African Republic OR Congo the Democratic Republic Sao Tome and Principe OR Burundi OR Eritrea OR Madagascar OR Reunion OR Somalia OR Comoros OR Ethiopia OR Rwanda OR Djibouti OR Kenya OR Mayotte OR Seychelles OR Uganda OR Mozambique OR Zambia OR Malawi OR Tanzania, Zimbabwe OR Benin OR Liberia OR Saint Helena OR Burkina Faso OR Gambia OR Mali OR Ghana OR Mauritania OR Senegal OR Cape Verde OR Cote D'ivoire OR Guinea OR Niger OR Sierra Leone OR Guinea-Bissau OR Nigeria OR Togo OR Algeria OR Egypt OR Libyan Arab Jamahiriya OR Morocco OR Tunisia OR Western Sahara OR Sudan) Filters applied: from 01/03/2010–31/03/2025. |
|  |  |
