# Supplementary material for: Burden and predisposing factors of physical inactivity among adults in Africa: Systematic review and Meta-analysis
Source: PLoS One. 2026 May 11;21(5):e0348786. doi: 10.1371/journal.pone.0348786 (PMC13160333; doi:10.1371/journal.pone.0348786)
Supplement: S2 Table — (DOCX) [file pone.0348786.s002.docx]

Supplementary Table2: Quality assessment for individual studies using Newcastle Ottawa quality assessment scale

| Author | Year of publication | Representativeness  Of the sample | Sample Size | Non Respondents | Ascertainment  Of outcomes | The Study Control for the most Important factor | The study control for any additional factor | Assessment of the out come | Statistical test | Total score |
| --- | --- | --- | --- | --- | --- | --- | --- | --- | --- | --- |
| Oyeyemi, Usman et al. | 2015 | * | * | * | ** | * | - | * | * | 8 |
| Msambichaka, Abdul et al. | 2018 | * | * | * | ** | * | - | * | * | 8 |
| Kirunda 2017 | 20117 | * | * | * | ** | * | - | * | * | 8 |
| Ezekwesili, Ononamadu et al. | 2016 | * | * | * | ** | * | - | * | * | 8 |
| Ukegbu, Ukegbu et al. | 2022 | * | * | * | ** | * | - | * | * | 8 |
| Mengesha, Roba et al. | 2029 | * | * | * | ** | * | - | * | * | 8 |
| Tekalegn, Solomon et al. | 2022 | * | * | * | ** | * | - | * | * | 8 |
| Amenu, Gelibo et al. | 2017 | * | * | * | ** | * | - | * | * | 8 |
| Ige, Owoaje et al. | 2013 | * | * | * | ** | * | - | * | * | 8 |
| Ugwuja, Ogbonna et al. | 2013 | * | * | * | ** | * | - | * | * | 8 |
| Odunaiya, Ayodele et al. | 2010 | * | * | * | ** | * | - | * | * | 8 |
| Owoeye, Osho et al. | 2013 | * | * | * | ** | * | - | * | * | 8 |
| Amuzie, Ajayi et al. | 2022 | * | * | * | ** | * | - | * | * | 8 |
| (Adegoke and Oyeyemi 2011 | 2011 | * | * | * | ** | * | - | * | * | 8 |
| Enyew, Nigussie et al. | 2023 | * | * | * | ** | * | - | * | * | 8 |
| Ogah, Madukwe et al. | 2013 | * | * | * | ** | * | - | * | * | 8 |
| Gichu, Asiki et al. | 2018 | * | * | * | ** | * | - | * | * | 8 |
| Oladapo, Salako et al. | 2010 | * | * | * | ** | * | - | * | * | 8 |
| Odugbemi, Onajole et al | 2012 | * | * | * | ** | * | - | * | * | 8 |
| Awotıdebe, Bısırıyu et al. | 2017 | * | * | * | ** | * | - | * | * | 8 |
| Abdeta, Teklemariam et al. | 2018 | * | * | * | ** | * | - | * | * | 8 |
| Shiferaw, Yalew et al. | 2024 | * | * | * | ** | * | - | * | * | 8 |
| Aliyu, Chiroma et al. | 2015 | * | * | * | ** | * | - | * | * | 8 |
| Oyeyemi and Adeyemi 2013 | 2013 | * | * | * | ** | * | - | * | * | 8 |
| Agaba, Akanbi et al. | 2017 | * | * | * | ** | * | - | * | * | 8 |
| Abd El Aty, M. A., et al | 2019 | * | * | * | ** | * | - | * | * | 8 |
| Farrag, N., et al. | 2019 | * | * | * | ** | * | - | * | * | 8 |
| El-Gilany etal. | 2011 | * | * | * | ** | * | - | * | * | 8 |
| Nketiah et al | 2023 | * | * | * | ** | * | - | * | * | 8 |
| Andersen, E., et al. (2021 | 2021 | * | * | * | ** | * | - | * | * | 8 |
| Ali, A. M. | 2022 | * | * | * | ** | * | - | * | * | 8 |
| Oyeyemi, A. L., et al. | 2015 | * | * | * | ** | * | - | * | * | 8 |
| Yousif, M. M., et al. | 2019 | * | * | * | ** | * | - | * | * | 8 |
| Malambo, P., et al. | 2016 | * | * | * | ** | * | - | * | * | 8 |

**Note: one * stands for 1 score**
